# Research on the transformation from international exhibition to “cloud” exhibition in the post COVID-19 era: A case study of China International Fair for Investment & Trade

**DOI:** 10.1371/journal.pone.0267455

**Published:** 2022-04-28

**Authors:** Ting Li, Qianqian Ye, Qiuxin Chen

**Affiliations:** 1 School of Internet Economics and Business, Fujian University of Technology, Fuzhou City, Fujian Province, China; 2 School of Economics and Management, Fujian Agriculture and Forestry University, Fuzhou City, Fujian Province, China; 3 Planning Department, Fujian Provincial Technology Transfer & Industrialization Service Co., Ltd, Fuzhou City, Fujian Province, China; Universidad Nacional Autonoma de Nicaragua Leon, NICARAGUA

## Abstract

By exploring the China International Fair for Investment and Trade’s development process, this study analyzes its absolute advantages in future development, gradually lost comparative advantages, and potential crises. Through the data envelopment analysis model, the study analyzes its resource allocation efficiency based on two cooperation modes: traditional “Offline” investment mode and “Online + Offline” investment mode. Then we use vector autoregressive model to comprehensively investigate the impact and causality of the two input factors on output. We find that its comprehensive allocation efficiency presents a “U” shape that reflects the characteristics of its three stages: the first stage, 2001–2005; the second stage, 2006–2012; and the third stage, 2013–2020. The main factors affecting resource allocation efficiency are then deduced from the results: exhibition scale utilization, booth design innovation, project strength of participating enterprises, and the signing rate of overseas customers. The number of industrial and commercial groups (X_4_) and participating countries and regions (X_6_) have an important impact on the output indicators: signed contract projects (Y_1_). The empirical results verify that the “Online + Offline” investment mode is an effective and suitable mechanism to solve the problem of cooperation and investment constrained by the COVID-19 pandemic. Based on the results of empirical analysis, this study posits the path and countermeasures of realizing the transformation to “cloud” exhibition in the post-pandemic era. Especially, we should focus on building new mechanisms of business environment that will promote the active participation of national, regional, and international industrial and commercial groups. The purpose is to continuously strengthen the foundation of cooperative trust and innovate a new trust model of online cooperative trading.

## 1. Introduction

The convention and exhibition industry is not only a “barometer” reflecting the development of a city but also a critical window showcasing the city’s image. Globally, cities such as Frankfurt, Zurich, and Barcelona are not only important business centers but also famous international convention and exhibition centers. Convention and exhibition economy is a new economic growth point [1, 2]. In 1987, China International Fair for Investment and Trade (CIFIT) set the stage for China’s opening to the outside world and government investment attraction created by Fujian. In 1993, it formed its own “golden key,” the earliest gateway for Chinese investment to the world market and world capital to the Chinese market. In 1997, CIFIT (held on September 8 every year; hereinafter also referred to as “9·8”) was upgraded to the first China investment and trade fair, becoming the largest international investment and trade conference in China.

The improvement of international exhibition competitiveness is conducive to the improvement of city competitiveness. The convention and exhibition industry can stimulate and drive the development of local cities (clusters). As a new industrial economy, it can optimize urban industrial structure and promote urban functional positioning. It has a strong radiation effect and urban marginal incremental effect [3, 4].

President Xi Jinping gave a great deal of affirmation in the congratulatory letter of the 20th CIFIT: The CIFIT, committed to building three platforms, two-way investment promotion, authoritative information release, and investment trend research, has developed into one of the most significant international investment events in the world [5]. While differing from Child Hill and Kim [6], Sassen [7] believes that the primary basis for the formation of global cities is investment globalization and financial securitization, which can create advanced production sites with high-level business services [6, 7]. This is the main function of the international exhibition platform. From the dynamic perspective of Taylor (2016) and O’Neill (2003), Su Ning (2010) proposed that the development process of China’s international cities should be distinguished from that of European and American countries, and innovation should be carried out according to the characteristics of regional cities [8–10]. As an international exhibition platform, CIFIT has the function of providing high-end services and investment globalization. From the perspective of historical development and practical development needs, it is an innovative economic platform for Fujian, a stage for investment cooperation among countries worldwide, and a world-class economic and trade incubator. It is an important platform for high-level opening to the outside world and plays a positive role in building an open world economy [5].

In 2020, the pandemic interrupted the orderly growth of the global economy. As a major international economic and trade activity held during the normalized pandemic prevention and control period, the 2020 CIFIT was held safely, orderly, and smoothly. While the offline scale was slightly reduced, its exhibition area still reached 110 000 square meters, and the conference still attracted 1018 business groups. The three-dimensional (3D) exhibition hall of “Cloud Investment Fair” made an outstanding appearance. During the conference, more than 2300 projects reached cooperation agreements, with a total investment of more than 800 billion RMB [11]. As an international investment and trade platform, this reflects CIFIT’s appeal and significant contribution to the regional economy.

In summary, scholars at home and abroad have performed a great deal of research and analysis on the promotion of regional economic growth through the convention and exhibition industry, mainly focusing on the qualitative analysis of the economic radiation of the exhibition. There is less quantitative analysis on the development mode and mechanism of the exhibition itself and its resource allocation, especially under the background of the pandemic situation. CIFIT has more than 20 years of history and has established a new pattern for world exhibitions. Therefore, this study focuses on the sustainable development mechanism resulting from its resource allocation efficiency. In particular, as a platform for international investment and trade, CIFIT has made notable contributions to the global economy against the background of the pandemic. There is no research on the resource allocation of the “Online + Offline” investment and trade model or the impact of this innovative model on the transformation and upgrading of international exhibitions during the pandemic. Therefore, this study focuses on this value of CIFIT.

## 2. CIFIT background

### 2.1 Analyzing absolute advantages of CIFIT

On June 25, 1985, Xiamen Fushan International Exhibition City was completed and held the “Xiamen International Exhibition,” ensuring that a modern economic and trade exhibition officially entered the historical stage of Fujian. On September 6, 1987, the “South Fujian Triangle Foreign Investment and Trade Fair” was hosted by the People’s Government of Fujian Province. In 1988, it was renamed “Fujian Investment and Trade Fair,” becoming a symbol of Fujian’s opening up and an important platform for mobilizing foreign investment. In 1997, the “Fujian Investment and Trade Fair” was upgraded to the “China Investment and Trade Fair” hosted by the Ministry of Commerce of the People’s Republic of China, leading to the rise and development of the exhibition industry in Fujian Province. Therefore, CIFIT has been held from 1997–2020. It has been called the “China Xiamen International Fair for Investment and Trade” since 2015, depending on the “big and small years.” In the big year, it is called the “China International Fair for Investment and Trade,” and in the small year, it is called the “China Xiamen International Fair for Investment and Trade.”

It can be observed from the development process of its 20th session that CIFIT still has the following absolute advantages:

First, from a world-class perspective, CIFIT is not an ordinary international exhibition but also a stage for global investment and trade cooperation. It is a world-class platform, which has changed the mode of world exhibitions and formed a comprehensive world-class investment and trade platform integrating exhibitions and conferences, a two-way investment mode. CIFIT, jointly sponsored by six major international economic organizations—including United Nations Conference on Trade and Development, United Nations Industrial Development Organization, World Trade Organization (WTO), Organization for Economic Co-operation and Development, International Finance Corporation of World Bank, and World Association of Investment Promotion Agencies—is not only the gateway of China’s opening to the outside world but also the largest international investment exhibition in the world. This makes its host city fully qualified to become an international urban center.

Second, from the perspective of uniqueness, CIFIT has introduced a new exhibition mode. Mr. Rubens Ricupero asked why so many international economic organizations jointly hold CIFIT—it has built a “harmonious platform” for the sustainable growth of the world economy [12]. This world-class platform established a new pattern for world exhibitions because, before CIFIT, there was no world-class comprehensive exhibition, no cooperation platform for trade and investment, and no cooperation platform for two-way investments from all countries in the world. This platform fully realizes the new mode of convention and exhibition, integrating exhibition and conference, and creates a “golden key” for a win-win situation.

Third, in terms of scale, more than 100 countries, industrial and commercial groups, and 31 provincial and municipal government investment delegations participated in CIFIT, which is the only fair in the country or even in the world with such scale of involvement. It can be said that during the CIFIT held in September, half the world was in Xiamen, Fujian, China. Countless agreements were signed, and more than 1000 billion RMB of global capital was prospected for projects. It is a world-class platform that innovates new international and regional cooperation mechanisms, such as international multilateral cooperation, regionally coordinated development, and volunteer service.

### 2.2 Analyzing the potential crises of CIFIT

#### 2.2.1 Not fully utilizing the advantage of “Xiamen-double high city”

According to the regulations of the international metropolis association, having a perennial world economic and trade exhibition is a necessary condition for being an international metropolis. Xiamen is not an ordinary international city. In 2016, Xiamen became the only Chinese city to host the Brazil, Russia, India, China, and South Africa (BRICS) summit. To this end, President Xi Jinping personally hosted the BRICS summit in Xiamen and highly appreciated Xiamen as a “two high” city (known as a high-quality and beautiful city). This is not only the “international business card” of the convention and exhibition city but also an important relationship between the “international convention and exhibition city” and the development of “world cities.” This “two high” comparative advantage of Xiamen city is both a new business mode and driving force for the future development of world cities. What role does Xiamen actively play in the division of labor in world economic growth? How can Xiamen play its due role as an international city with higher quality? Using a world-class exhibition to promote Xiamen’s development into a world-class city in the new era is, thus, a strategic choice.

#### 2.2.2 Facing the catch-up problem of other major domestic exhibitions

In order to realize the leap to an international metropolis, Xiamen should make use of the world-class stage of CIFIT as a necessary condition, open this public service platform to the world to a much greater extent, and work with the six major economic organizations to build more and more key mutual projects. In this regard, both Xiamen and CIFIT have great development potential. However, it can be seen from the current development situation that the exhibitions in neighboring provinces have many advantages and strong characteristics: strong integration, a considerable pace of internationalization, and a high level of systematization, which virtually weakens the absolute advantage of CIFIT and brings immense catch-up pressure. If the existing “absolute advantage” is not consolidated, it could face the problem of “abandonment of the market.” Simultaneously, the administration of the endogenous organization, the flattening of the endogenous mechanism, and the normalization of the endogenous power of CIFIT, have held a double-edged sword to its further development, which is not only the current double pressure but also the driving force of subsequent innovation.

#### 2.2.3 Five mismatches of CIFIT

In the 20-year span of the development process, CIFIT has had several mismatches in its status and role: urban positioning and regional integrated development; the scale of regional GDP and the positioning of the world’s largest investment exhibition; the international investment functions and the degree of platforms into the six international organizations; the efficiency of expected resource input and expected output; and the pattern of coordinating management institutions and a world-class investment service platform. Particularly, there exists the problem of organizational flattening, the fact that local enterprise groups cannot absorb and digest the resources of international organizations in coordination, and the imbalance between the level of international organizations and local coordination institutions. This needs to be further improved and innovated through the government’s macro-control to gradually form endogenous new advantages and provide new momentum for CIFIT. It could promote Xiamen to achieve higher quality development and gradually develop into a world-class city.

### 2.3 Using the advantage of the Maritime Silk Road core area

Fujian is not an ordinary coastal province. As early as the Song and Yuan Dynasties, Fujian Erythrina port was the world’s largest trade port and the starting point of the ancient Maritime Silk Road. In the Qing Dynasty, Xiamen and Fuzhou became part of the five trading ports. During the reform and opening-up period, Fujian and Guangdong became special zone provinces that “adopted special opening and flexible measures.” In 2015, Fujian Province was approved as the “core area of the 21st century Maritime Silk Road.” Nowadays, the international urban belt and bay area are booming. As the core area of the Maritime Silk Road, the new Fujian gets a second chance by taking advantage of the international economic and trade stage of CIFIT. Transforming the sustainable growth mechanism of the world economy into the belt and road investment and trade public service platform is another strategic choice.

According to the analysis of the development status, potential crises, and mismatches of CIFIT, although CIFIT has innovated a new cooperation mechanism of international multilateral cooperation, its potential crises and several major mismatches are important factors hindering the sustainable development of this world-class international convention and exhibition platform.

This study makes a quantitative analysis of its internal resource allocation efficiency by constructing a data envelopment analysis (DEA) model and proposes some suggestions to promote the transformation and development of CIFIT in the post-COVID-19 pandemic era.

## 3. Methodology

### 3.1 Construction of the DEA model

DEA is a method proposed by Charnes, Cooper, and Rhodes [13] to evaluate the output. It involves the use of linear programming methods to construct a non-parametric piecewise surface or frontier over the data to enable the calculation of efficiencies relative to the surface. It is mainly used to evaluate the relative efficiency model of the multi-factor productivity of homogeneous decision-making unit (DMU) groups. Coelli and Rao and Zuniga González used DEA to derive Malmquist productivity indices [14, 15]. González (2011) applied frontier production function analysis to small farms in Nicaragua during 1998–2005. In their case, an average firm was an agricultural production unit [16]. Coelli and Rao proposed that the two measures provided the same technical efficiency (TE) scores when a constant returns to scale (CRS) technology was applied but are unequal when variable returns to scale (VRS) was assumed. They believed that the choice of orientation was not a big issue in this case. An output orientation was selected in their study because it would be fair to assume that, in agriculture, one usually attempted to maximize output from a given set of inputs, rather than the reverse [14]. The CCR model is based on the assumption of CRS, and the BCC model is based on the assumption of VRS. Charnes et al. defined “relative efficiency” as “the ratio of output to input” in the form of “ratio” [17, 18]. Dios-Palomares proposed that the assessment of environmental efficiency was also related to productivity and measured efficiency with frontier methods including environmental variables [19]. This study applies two main DEA models: CCR and BCC. An input orientation is chosen in this study and for every year of its production unit.

This study uses the DEA model to determine the effective production frontier by analyzing sample input and output data and determining whether each DMU is DEA effective according to the distance between each DMU and the production frontier. The advantage of the DEA method is that it can deal with multi-input and multi-output problems. It can not only judge whether the corresponding point of DMU is located on the effective production frontier by linear programming but also obtains a lot of valuable management information. Therefore, this study analyzes the efficiency between the input and output of CIFIT with the help of the DEA method and searches the path that can improve the efficiency of resource allocation and promote the transformation of the International Exhibition innovation platform to a “Cloud Exhibition” platform.

It is assumed that there are t similar parts evaluated, which are called decision units:

$$h_{j}=\frac{u^{T}y_{j}}{v^{T}x_{j}}=\frac{\sum_{r=1}^{n}u_{r}y_{rj}}{\sum_{i=1}^{m}v_{i}x_{ij}},j=1,2\ldots \ldots \text{t}$$

$$x_{j}={\left(x_{1j},\ldots ,x_{mj}\right)}^{T},y_{j}={\left(y_{1j},\ldots ,y_{nj}\right)}^{T},j=1,2,\ldots ,t\;h_{j}\leq 1,j=1,2,\ldots ,t$$

The slack variable is introduced in the following model:

$$(D_{CCR})\{\begin{array}{c} \text{min}\theta \\ s.t.\sum_{j-1}^{t}\lambda_{j}x_{j}\text{+}S^{-}=\theta x_{0}\\ \sum_{j=1}^{t}\lambda_{j}y_{j}-S^{+}=y_{0}\\ \lambda_{j}\geq 0\\ S^{+}\geq 0,S^{-}\geq 0 \end{array}$$

*x*_0_ and *y*_0_ represent input and output vectors for each year, respectively; *x*_*j*_ and *y*_*j*_ represent the input and output vectors produced in year *j*; *S*^+^ refers to the slack variable of output, and *S*^−^ is the residual variable of input; *λ*_*j*_ represents the weight of each DMU; *θ* represents the overall efficiency under the assumption of fixed scale income, which is between 0 and 1, reflecting the input-output efficiency of the exhibition’s annual production. If *θ* = 1, it means that the DMU is DEA effective and indicates that the input-output is completely effective; that is, both TE and scale efficiency (SE) are effective. TE means that the exhibition platform can completely use current resources to achieve maximum output and optimal operating conditions. The efficiency of scale means that the production of the exhibition platform is in the stage of fixed returns to scale; that is, the output and input are expanded or reduced in the same proportion. If *θ* < 1, DMU and DEA are invalid, it means that the existing use of technology and production factor allocation are not in the best state.

In 1988, Banker, Charnes, and Cooper decomposed TE into pure technical efficiency (PTE) and SE, and TE = PTE * SE. By adding constraints on weight, they established the following VRS model [20]:

$$(D_{BCC})\{\begin{array}{c} \text{min}\theta \\ s.t.\sum_{j-1}^{t}\lambda_{j}x_{j}\leq \theta x_{0}\\ \sum_{j=1}^{t}\lambda_{j}y_{j}\geq y_{0}\\ \sum_{j-1}^{t}\lambda_{j}=1\\ \lambda_{j}\geq 0,j=1,2,\ldots ,t \end{array}$$

The conversion relationship between PTE and SE can further measure the SE of each DMU. It can also estimate whether the annual production scale is best under the condition of fixed input. The CCR model is applied to measure the total efficiency when DMU is in a fixed returns to scale. Fixed scale compensation is the efficiency evaluation of all DMUs compared together. The BCC model is applied to DMUs in the case of VRS, which is used to measure pure technology and SE and compare the VRS with rated units with equivalent conditions.

### 3.2 Index system

In the specific selection of DMU indicators, the number must be larger than the product of the number of input and output indicators and at least twice the sum of the two [2]. The establishment of the index system in this study is shown in Table 1. The DMU is the CIFIT session. The output indicators are Y_1_ and Y_2_. Y_1_ represents the signed contract projects, and Y_2_ represents the amount of contracted foreign capital. These two indicators directly and intuitively reflect the effectiveness of the international platform function of CIFIT in stimulating investment and trade. There are six input indicators of the relevant absolute advantage variables extracted from the development history of the 20th CIFIT: X_1_ represents the exhibition area, X_2_ represents the exhibition booths, X_3_ represents the number of participating enterprises, X_4_ represents the number of industrial and commercial groups, X_5_ represents the number of media participants, and X_6_ represents the number of the participating countries and regions.

**Table 1:** Allocation efficiency index system of CIFIT.

	Variable	Variable description	Unit
**Output indicators**	**Y**_**1**_	Signed contract projects	Individual
	**Y**_**2**_	Amount of contracted foreign capital	USD100mn
**Input index**	**X**_**1**_	Exhibition area	square meter
	**X**_**2**_	Exhibition booth	Individual
	**X**_**3**_	Number of participating enterprises	Individual
	**X**_**4**_	Number of industrial and commercial groups	Individual
	**X**_**5**_	Number of media participants	Individual
	**X**_**6**_	Participating countries and regions	Individual

### 3.3 Data source

The data for this study are mainly from official websites such as the China Statistical Yearbook, China Exhibition Yearbook, CIFIT, the release of previous achievements, invitations, conference guidelines of CIFIT, Xiamen Daily, and other authoritative media units. Some missing data are interpolated, and the final data used for model analysis are obtained through sorting and calculation. In 1997, CIFIT was upgraded from the provincial to the national level, becoming the first national-level fair. In 2001, the fifth CIFIT successfully held 30 two-way investment seminars. This provided a convenient way for Chinese enterprises to understand the overseas investment environment and a suitable environment for investment negotiations. The year 2001 was an important turning point for China to integrate into the world and for the world to understand China. In the process of data verification, this study mainly observes the investment and trade situation of the CIFIT platform after China acceded to the WTO.

This study primarily selected data after 2000. At the same time, due to the lack of some variable data of the four sessions from 1997 to 2000, the data for these four years were excluded. Finally, the 20 year data from 2001 to 2020 were used in the model. In particular, 2020 is a special year: all offline activities were mostly suspended due to the pandemic, which created great difficulties for data collection. Therefore, the output index data mainly came from CIFIT’s official data. The input index **X**_**3**_ (the number of participating enterprises) is calculated from official statistics. The data of the previous sessions are mainly calculated by sorting out the relevant data, such as the number of multinational enterprises participating in the conference, the number of enterprises participating in the negotiation, and the number of successful docking enterprises published in the official media. The data on some indicators are extracted from the information released by the official media, and the missing years of some indicators are interpolated. Specifically, the variable **X**_**3**_ of the number of participating enterprises has two missed data instances in 2002 and 2006. The data cannot be collected from the official website, report, or newspaper during these 2 years. We used interpolation by averaging these 2 years. Then, we interviewed the relevant professors of government departments who took part in organizing the CIFIT before. We obtained an approximate number that is similar to interpolation in Table 2. All data are listed as shown in Table 2.

**Table 2:** Resource input and output index data of CIFIT.

Year	Y_1_	Y_2_	X_1_	X_2_	X_3_	X_4_	X_5_	X_6_
	Signed contract projects	Amount of contracted foreign capital	Exhibition area	Exhibition booth	Number of participating enterprises	Number of industrial and commercial groups	Number of media participants	Participating countries and regions
**2001**	1027	47.98	28000	2500	500	117	1100	97
**2002**	1151	51.91	28000	2500	460	141	1032	96
**2003**	1259	66.52	28000	2500	421	240	1200	102
**2004**	1110	81.22	33000	2500	520	300	1296	118
**2005**	1053	122.43	33000	2500	474	350	1393	125
**2006**	752	76.17	33000	2500	731	383	1389	113
**2007**	669	96.2	33000	2500	1053	414	1385	119
**2008**	517	80.99	52000	2500	1246	445	1392	126
**2009**	515	75.3	60000	2800	1157	492	1400	125
**2010**	484	99.6	65000	3214	1378	627	1439	144
**2011**	339	104.6	65000	4000	1600	636	1478	112
**2012**	355	154.2	100000	4000	2200	650	1500	132
**2013**	1386	321.6	100000	4000	2870	556	1500	118
**2014**	1455	336.4	100000	4000	3000	603	1500	126
**2015**	1571	352.9	100000	4000	4000	650	1500	105
**2016**	1502	349.34	138000	6000	5000	707	1300	104
**2017**	1577	366.73	120000	5000	4040	700	1000	107
**2018**	1982	365.2	130000	6000	5000	1005	1500	128
**2019**	2100	375.4	130000	6000	3000	1008	1500	130
**2020**	2300	307.5	110000	5000	2000	1018	700	69

## 4. Results and discussion

Based on the DEA model above, the resource allocation efficiency of CIFIT is analyzed based on the empirical results. From the input indicators, it can be directly seen that during the pandemic period in 2020, the offline exhibition area of CIFIT reached 110 000 square meters. At the same time, the 3D exhibition hall of “cloud CIFIT” made a significant appearance in time, which opened a new model. In terms of input indicators of the year 2020, the number of X_1_ area, X_2_ booth, X_3_ participating enterprises, X_5_ participating media, and X_6_ participating countries and regions decreased sharply compared with 2019, especially the number of X_6_. Since the pandemic is global, it directly reduces exchanges between countries. It can be seen from the output indicators that the amount of foreign capital used in contracts (Y_2_) decreased, but the number of contract projects signed (Y_1_) increased, as shown in Table 2 [21–25]. In theory, most indicators should fall, but why did contracts increase? After an increased online investment, we assume that the offline area can be more optimized, and a better new format of digital exhibition should be designed. This study takes 2020 as a watershed and divides the empirical analysis into two parts to illustrate the efficiency of allocation of CIFIT: (1) select 2001–2019 as DMUs to estimate the resource allocation efficiency of CIFIT under the traditional “Offline” investment trading mode and (2) select 2001–2020 as DMUs to estimate the resource allocation efficiency of CIFIT under the “Online + Offline” investment cooperation mode spawned under the COVID-19 pandemic.

### 4.1 Traditional “Offline” investment cooperation mode

According to the above model, the study selected 19 DMUs each year from 2001 to 2019 and estimated the resource allocation efficiency of CIFIT using the DEAP 2.1 software on the input and output data in Table 2. The results are shown in Table 3.

**Table 3:** Resource allocation efficiency of CIFIT from 2001 to 2019.

Year	Comprehensive efficiency (crste^a^)		Pure technical efficiency (vrste^b^)		Scale efficiency (scale^c^)		
	Value	Effectiveness	Value	Effectiveness	Value	Effectiveness	Returns to scale
2001	1.000	√	1.000	√	1.000	√	-
2002	1.000	√	1.000	√	1.000	√	-
2003	1.000	√	1.000	√	1.000	√	-
2004	0.930		1.000	√	0.930		Increasing
2005	1.000	√	1.000	√	1.000	√	-
2006	0.687		1.000	√	0.687		Increasing
2007	0.788		1.000	√	0.788		Increasing
2008	0.505		1.000	√	0.505		Increasing
2009	0.447		0.893		0.501		Increasing
2010	0.491		0.820		0.599		Increasing
2011	0.488		0.870		0.561		Increasing
2012	0.553		0.769		0.719		Increasing
2013	1.000	√	1.000	√	1.000	√	-
2014	1.000	√	1.000	√	1.000	√	-
2015	1.000	√	1.000	√	1.000	√	-
2016	0.980		1.000	√	0.980		Increasing
2017	1.000	√	1.000	√	1.000	√	-
2018	0.968		0.973		0.994		Increasing
2019	1.000	√	1.000	√	1.000	√	-
Mean	0.834		0.964		0.856		

^**a**^Crste = technical efficiency from CRS DEA.

^**b**^Vrste = technical efficiency from VRS DEA.

^**c**^Scale = scale efficiency = crste/vrste.

Crste is the TE (comprehensive efficiency) without considering the returns to scale; Vrste is the TE (PTE) when considering the returns to scale; scale refers to the SE when considering the returns to scale. PTE and SE are the subdivision of comprehensive efficiency. When the crste evaluation value is 1, the DMU in this year is scale effective and technology effective [26].

In terms of comprehensive efficiency, the average resource allocation efficiency of 19 years from 2001 to 2019 was 0.834, of which 9 years had comprehensive efficiency of one and were technically effective. Except for these nine DMUs, the comprehensive efficiency in other years did not reach the best. The lowest year was 2009, whose comprehensive technical efficiency is 0.447 (as shown in Table 3). Even the comprehensive allocation efficiency in 7 years from 2006 to 2012 presents a U-shape, falling first and then rising.

In terms of PTE, the average PTE in 19 years from 2001 to 2019 is 0.964, of which 14 years, whose PTE is one, are technically effective. This shows that the pure DEA technology of CIFIT is effective. It also means that CIFIT basically runs effectively, and the management level is also relatively effective. The PTE is greater than SE, which shows that SE is the main reason for the low comprehensive efficiency, and the problem is that the resource allocation scale does not match the optimal scale.

In terms of SE, the average SE in 19 years from 2001 to 2019 is 0.856. Similarly, the lowest year is 2009, with an SE of 0.501 and PTE of 0.893 (as shown in Table 3). These are the dual reasons for the low comprehensive technical efficiency in 2009. Although the scale of resource allocation does not match the optimal scale, the return to scale is increasing.

The overall resource allocation of CIFIT can be divided into three stages (as shown in S1 Fig).

The first stage is 2001–2005, the second stage is 2006–2012, and the third stage is 2013–2019. It can be seen directly that the comprehensive efficiency of 2001–2003, 2013–2015, 2017, and 2019 are effective, the resource allocation efficiency of these nine sessions is much higher, and the output capacity is stronger given the input resources. In the first stage, the comprehensive efficiency is basically effective, the PTE is effective, and the SE is above 0.93.

The second stage is the year group with a large range. The comprehensive efficiency of the years 2006–2012 is not effective. The basic problem is the SE. The overall resource allocation of CIFIT showed a U-shaped trend from the effective, comprehensive efficiency in 2005 to the lowest comprehensive efficiency in 2009, and then to the effective basic comprehensive efficiency after 2013. From 2006 to 2008, the technical efficiency is effective, and the return to scale is not effective, but the return to scale of resource allocation is increasing. From 2009 to 2012, both technology efficiency and SE are not effective, but the returns to scale of resource allocation are increasing. The result shows that the SE of CIFIT has been rising, meaning that the whole CIFIT international exhibition is in the state of SE.

The third stage, 2013–2019, presents the effective results of comprehensive efficiency every other year. Except for 2018, the PTE of all years in this stage is effective, and the SE is above 0.98 (as shown in Table 3). This is related to the alternating holding of “CIFIT” and “Xiamen CIFIT” and the implementation of the system of large and small years; the overall efficiency fluctuates in the same direction with a lag. Comprehensive inefficiency occurred in 2016 and 2018; however, for the former, this was not true for scale. In 2018, both technology and scale were not effective, so the scale had to be expanded accordingly.

From the solution of the BCC model, the slack variables and redundant variables of each DMU in the second stage, 2006–2012, can be obtained. The situation was similar in 2006, 2007, and 2008. The PTE of CIFIT resource allocation was effective, but the SE was ineffective. This mainly reflected that the output Y_1_ (the number of contracted projects) did not achieve the expected benefits, and the slack variable values were 471.445, 480.644, and 609.845, respectively (seeing data in S1 Text). It is related to the mismatch between the current situation of the sudden increase in the exhibition area of X_1_, the number of participating enterprises in X_3_, and the decline in the number of contracted projects in Y_1_.

The situation of the years 2009, 2010, 2011, and 2012 is similar. The PTE was invalid, and the SE was also ineffective. The slack variable values are 646.391, 676.082, 874.180, and 937.697, respectively (seeing data in S1 Text). It is related to the mismatch between the exhibition area of X_1_, the exhibition booth of X_2_, the number of participating enterprises of X_3_, the sudden increase of X_4_ industrial and commercial groups, and the decline in the number of contracted projects in Y_1_. Accordingly, there were non-zero redundant variables in these 4 years. In order to make DEA effective, under the condition that the output indicators remain unchanged, outstanding indicators were extracted for observation from the results. For instance, 300 booths should be reduced in 2009. In 2010, about 579 booths should be reduced, and the number of participating media should be reduced by about 259(seeing data in S1 Text). In 2011, the scale of participating countries and regions did not match the output. Based on the existing output, 97–98 participating countries and regions should be matched. However, the actual situation is that 112 participating countries and regions participated in CIFIT. Since the resource conditions of this variable input were not fully explored, the output of the actual number of signed contracts and the amount of contracted foreign capital utilization was less than expected. In 2012, the exhibition hall area expanded from 65 000 square meters to 100 000 square meters (as shown in Table 3). Under the condition of unchanged output indicators, about 923 booths should be reduced, and the number of domestic and foreign media participants should be reduced by 346(seeing data in S1 Text). However, this does not mean reducing the scale. It can be obtained from the increasing return on the scale of resource allocation from 2006 to 2012. Instead, the results mean that various innovative measures should be taken to improve efficiency and increase output.

The development of phase III, 2013–2019, is consistent with China’s Belt and Road Initiative. Many forums were held in CIFIT around the theme of the Belt and Road Initiative. Compared with the input indicators in the second stage, in addition to the sharp increase of X_4_ industrial and commercial groups, X_1_ exhibition area, X_2_ exhibition booth, X_5_ number of participating media, and X_6_ participating countries and regions, these indicators exhibited little change, and all investment indicators tended to be stable; even the domestic and foreign media institutions decreased slightly. However, compared with the output index Y_1_ of the second stage, the average growth rate of the third stage is 2.57 times that of the second stage. Compared with the output index Y_2_ of the second stage, the average growth rate of the third stage is 2.76 times that of the second stage. This presents the effective result of the overall comprehensive efficiency of the third stage.

The most significant feature of the third stage was that comprehensive efficiency was effective every other year, which is related to the alternating holding of CIFIT. In these years, the comprehensive efficiency evaluation is not one, but the SE value is still increasing, which shows that CIFIT should make full use of the existing invested resources to increase output, establish a mechanism of investment without big or small years, innovate the function of fixed normalization platform, and realize the optimal allocation of resources.

### 4.2 “Online + Offline” investment cooperation mode

According to the above model, this study selected 20 DMUs each year from 2001 to 2020 and used DEAP 2.1 software to estimate the resource allocation efficiency of CIFIT based on the input and output data in Table 2. We added the 2020 data to observe whether the pandemic had a profound impact on CIFIT. We also attempted to assess whether online cooperation and investment mode affect CIFIT. The results are shown in Table 4.

**Table 4:** Resource allocation efficiency of CIFIT from 2001–2020.

Year	Comprehensive efficiency (crste^a^)		Pure technical efficiency (vrste^b^)		Scale efficiency (scale^c^)		
	Value	Effectiveness	Value	Effectiveness	Value	Effectiveness	Returns to scale
2001	1.000	√	1.000	√	1.000	√	-
2002	1.000	√	1.000	√	1.000	√	-
2003	1.000	√	1.000	√	1.000	√	-
2004	0.917		1.000	√	0.917		Increasing
2005	1.000	√	1.000	√	1.000	√	-
2006	0.687		1.000	√	0.687		Increasing
2007	0.788		1.000	√	0.788		Increasing
2008	0.488		1.000	√	0.488		Increasing
2009	0.429		0.893		0.481		Increasing
2010	0.485		0.820		0.592		Increasing
2011	0.480		0.793		0.606		Increasing
2012	0.540		0.769		0.702		Increasing
2013	1.000	√	1.000	√	1.000	√	-
2014	1.000	√	1.000	√	1.000	√	-
2015	1.000	√	1.000	√	1.000	√	-
2016	0.971		0.981		0.989		Increasing
2017	1.000	√	1.000	√	1.000	√	-
2018	0.868		0.939		0.924		Decreasing
2019	0.947		1.000	√	0.947		Decreasing
2020	1.000	√	1.000	√	1.000	√	-
Mean	0.830		0.960		0.856		

^**a**^Crste = technical efficiency from CRS DEA.

^**b**^Vrste = technical efficiency from VRS DEA.

^**c**^Scale = scale efficiency = crste/vrste.

As shown in Table 4, in the second estimation, the overall resource allocation of CIFIT can still be divided into three stages. Similar to Table 3, the comprehensive efficiency is effective in 9 years.

The first stage is 2001–2005, the second stage is 2006–2012, and the third stage is 2013–2020. The mean value of comprehensive efficiency and PTE decreased, but the SE remained unchanged.

As shown in S2 Fig, there are three main differences between these two estimates.

In the first estimate, the DEA is effective in 2019 (the Crstes is 1), but in the second estimate, the comprehensive efficiency and SE decline in 2019, and the DEA in 2019 is invalid. This shows that in the traditional model, the resource allocation in 2019 is DEA effective, but it does not achieve effective resource allocation in the innovative model.

Second, under the comparison of the “Online + Offline” innovation mode, the SE showed a decreasing trend in 2018 and 2019. In particular, the PTE in 2018 was 0.939 (as shown in Table 4), which did not achieve the optimal management mode and the optimal use of innovative technologies.

Third, in the second estimation, the PTE and SE are generally lower than the first estimation. This shows that the DEA of resource allocation efficiency in 2020 is effective despite the pandemic’s impact. The resource allocation of the “Online + offline” investment and trading mode is better than the traditional one. To some extent, this is a paradigm shift.

As shown in Table 4, from the solution of BCC model, in order to make DEA effective, for instance, in 2018, the exhibition hall area should be reduced by 7985.602 square meters, about 368 booths should be reduced, and the number of domestic and foreign media and participating countries and regions should be reduced by 92 and 8, respectively, under the condition that the output index remains unchanged(seeing data in S1 Text). This shows that under the conditions of constant input, the media at home and abroad and the resources of participating countries and regions were not fully utilized, resulting in less output. The SE decreases in 2018 and 2019, which is related to the alternating holding of “CIFIT” and “Xiamen CIFIT.” In addition to the mismatch between the resource allocation scale and the optimal scale, there are also reasons for insufficient optimization in technology and management, which makes the comprehensive efficiency ineffective. It further shows that the transformation and upgrading of CIFIT should have been achieved starting from 2018 to achieve better efficiency of scale.

Under different development modes, CIFIT has expanded from the exhibition era of 1.0 in the initial stage to the exhibition era of 2.0 in the medium term. The current “Online + Offline” mode has opened the “digital era” of the exhibition; especially during the pandemic, cooperating with the tech giant Alibaba Group, the new online CIFIT App is the most up-to-date online international investment promotion platform built by the CIFIT Organizing Committee. The Organizing Committee opened the “Cloud Investment Fair,” creating a new investment promotion service mode. “Cloud CIFIT” has fully adopted 3D, artificial intelligence, cloud computing, big data, and other technologies; launched cloud display, cloud docking, cloud negotiation, cloud discussion, and cloud signing (five clouds), and set up cloud exhibition halls and 3D exhibition halls for 37 provinces, cities, autonomous regions, province-level municipality, and 38 countries and regions in China. The objective is to enable global businesses to overcome time and space constraints and negotiate and interact on a wider range and at a deeper level. At the same time, through platform data specialization, refined analysis, and intelligent algorithms, “Cloud CIFIT” could accurately match the investment and financing parties such that investors at home and abroad can obtain investment information with one click [11]. In 2020, the PTE reached DEA effectiveness, and the advanced technology and management system had been fully adopted such that the investment transactions that cannot be realized in offline space can be realized through a new digital model. Therefore, from the output data Y_1_, the number of contracts in 2020 did not decrease but instead increased because the demand for cooperation could be realized and completed online. However, the amount of contracted foreign capital Y_2_ needs to be implemented offline, so the amount is slightly less than that of the previous year.

Although the “cloud mode” of the exhibition is passively held due to the pandemic, it is also an innovative interpretation of the “Online + Offline” mode. Through DEA data analysis, this mode is DEA effective, which provides a good theoretical basis for exploring the “Cloud Exhibition” of various offline international exhibitions.

### 4.3 Results of the VAR model

Based on the analysis of DEA, the *number of participating enterprises (*X_3_), *industrial and commercial groups (*X_4_), and *participating countries and regions (*X_6_) has an important impact on the two output indicators: *signed contract projects (*Y_1_) and *amount of contracted foreign capital (*Y_2_). During the pandemic, the two variables (X_4_ and X_6_) had a direct and significant impact on the number of offline trading contracts. The vector autoregressive (VAR) model uses endogenous variables to regress the lag term of all endogenous variables of the model to estimate the dynamic relationship of all endogenous variables.


$$Y_{t}=c+\Pi_{1}Y_{t-1}+\Pi_{2}Y_{t-2}+\ldots +\Pi_{k}Y_{t-k}+u_{t},$$

$$Y_{t}=(y_{1,t}\;y_{2,t}\;\ldots \;y_{N,t})\prime ,\;c=(c_{1}\;c_{2}\;\ldots \;c_{N})\prime$$

$$\Pi j=[\begin{array}{cccc} \pi_{11.j} & \pi_{12.j} & \cdots & \pi_{1N.j}\\ \pi_{21.j} & \pi_{22.j} & \cdots & \pi_{2N.j}\\ \vdots & \vdots & \ddots & \vdots \\ \pi_{N1.j} & \pi_{N2.j} & \cdots & \pi_{NN.j} \end{array}],\;j=1,2,\ldots ,k$$

$$u_{t}=(u_{1,t}\;u_{2,t}\;\ldots \;u_{Nt})\prime$$

***Y***_***t***_ is the column vector of endogenous variables, ***Y***_***t-k***_ is the lag term of ***Y***_***t***_, *k* is the lag order of endogenous variables, *t* is the number of samples, ***Π***_***k***_ is the coefficient matrix, ***u***_***t***_ is the disturbance term, and *c* is the constant term.

X_4_ and X_6_ are important variables of offline exhibitions, but we should pay attention to and understand the impact on Y_1_ in the future after the combination of online and offline. This is very important for improving the “Online + offline” exhibition mechanism. Therefore, this study chooses Y_1_, X_4_, and X_6_ to enter the VAR model and uses the VAR model to further empirically analyze the impact of the change of X_4_ and X_6_ on Y_1_. Then, *Y*_*t*_ = (*Y*_1_, *X*_4_, *X*_6_), *t* = 1, 2,…, *T*.

We used Eviews 8 for regression analysis of the model. In order to avoid pseudo regression in regression analysis, it is necessary to test the stationary position of the following three variables: X_4_, X_6_, and Y_1_. Here, the unit root (ADF) of the three variables is tested.

It can be seen from Table 5 that Y_1_, X_4_, and X_6_ are stable after taking the first-order difference. We then built a three variable VAR model. The AR roots table is used to test the stability of the model. As shown in S3 Fig, the characteristic roots of all sequences are located in the unit circle, and the whole model is stable.

**Table 5:** ADF unit root test results.

Variable	Trend and intercept (P value)	Intercept (P value)	None (P value)	Result
**Y**_**1**_	0.8370	0.7675	0.8010	Unstable
**X**_**4**_	0.1871	0.0427	0.9980	Unstable
**X**_**6**_	0.8594	0.1202	0.4989	Unstable
**ΔY**_**1**_	0.0425	0.0163	0.0010	Stable
**ΔX**_**4**_	0.0376	0.0294	0.0135	Stable
**ΔX**_**6**_	0.0105	0.0158	0.0007	Stable

Next, we determined the lag order and model stability test. The optimal lag order of the model was determined according to LR, FPE, AIC, SC, and HQ [27]. It can be seen from Table 6 that the order selected by most information criteria is 4. The VAR model adopts lag order 1 for modeling.

**Table 6:** VAR lag order selection criteria.

Lag	LogL	LR	FPE	AIC	SC	HQ
0	-10.89140244	NA	0.001029	1.634283	1.78132	1.648898
1	24.46079663	54.06807*	4.76e-05*	-1.465976	-0.877825*	-1.407513
2	33.96455652	11.18089	5.10E-05	-1.525242*	-0.495978	-1.422931*
3	42.94717587	7.397451	7.36E-05	-1.523197	-0.052821	-1.377039

* Indicates lag order selected by the criterion.

LR: sequential modified LR test statistic (each test at 5% level).

FPE: Final prediction error.

AIC: Akaike information criterion.

SC: Schwarz information criterion.

HQ: Hannan-Quinn information criterion.

The purpose of the Granger causality test is to verify that there is not only a trend correlation between the two sequences, but also that the independent variable has a certain ability to explain and predict the future changes of the dependent variable. If the value of the test statistic exceeds the critical value at a certain significance level, the explanatory variable should be included in the model. It is called the Granger causality of the explained variable. This study focuses on whether X_4_, and X_6_, have the ability to explain and predict Y_1_. Also, we should know whether Y_1_ greatly influenced X_4_ and X_6_. Table 5 shows the results of the Granger causality test.

As shown in Table 7, X_4_ and X_6_ are Granger causality for Y_1_. It means that X_4_ and X_6_ are very important to Y_1_ However, it is easier to sign a contract successfully offline, which shows that “Online + Offline” mode should focus on how to establish a strong contact trust mechanism to strengthen the signing rate of online exhibitions. X_6_ and Y_1_ are not Granger causality for X_4_. X_6_ and Y_1_ could not affect X_4_. X_4_ is not a Granger reason for X_6_, but Y_1_ is a Granger reason for X_6_. The results show that the rate of signed contract projects will further affect the participation of countries and regions.

**Table 7:** Granger causality test results.

**Dependent variable: Y1**			
Excluded	Chi-sq	df	Prob.
X4	8.490720417	2	1.43E-02
X6	8.573306631	2	1.38E-02
All	10.32387439	4	0.035311521
**Dependent variable: X4**			
Excluded	Chi-sq	df	Prob.
Y1	0.904901999	2	0.636067239
X6	0.813556294	2	0.665791882
All	4.046243325	4	0.39978367
**Dependent variable: X6**			
Excluded	Chi-sq	df	Prob.
Y1	6.836787839	2	0.032765016
X4	0.98905838	2	0.609857967
All	7.402049246	4	0.11610688

In order to further study the dynamic relationship between X_4_, X_6_, and Y_1_ against the background of the pandemic, the study attempts impulse response to analyze the short-term dynamic relationship between the two systems. The impulse response results are shown in S4 Fig.

The impact of X_4_ on Y_1_ shows a wave rise. The impact of X_6_ on Y_1_ shows the characteristics of the cycle. After reaching the maximum in phase 5, it gradually decreases. The impact of X_4_ and X_6_ on Y_1_ does not show an immediate response. The response value in phase 1 was 0, and then decreased and increased slowly. The response value of X_4_ to itself shows a downward trend. The impact of X_6_ participating countries and regions on itself fell rapidly in the first phase, and then fluctuated around 0. The response of X_4_ on to X_6_ gradually weakened. The response of X_6_ to X_4_ rose rapidly in the first period then fluctuates repeatedly and gradually tends to 0.

Above all, the impact of X_4_ and X_6_ on Y_1_ is obvious. Among them, the impact of X_4_ on Y_1_ increases gradually. This shows that in the “Online + Offline” mode, we need to pay full attention to the power of X_4_ and the participation of countries and regions.

In order to analyze the importance of different variables in the process of variable prediction, we further decompose the variance.

As shown in Table 8, the contribution of X_6_ participating countries and regions to Y_1_ is 0 in phase 1, then slowly increases and then decreases, reaching a maximum of 32.25056% in phase 7. The contribution of X_4_ to Y_1_ is lower than that of X_6_, up to about 3.235723% in phase 2, and then decreases gradually. This shows that X_6_ plays a very important role in the signing rate of contract. This provides important theoretical support for how to improve countries and regions’ online participation and improve the cooperation-signing rate. Due to the natural advantages of offline signing, how to improve the “Online + Offline” mode and online cooperation system is of positive significance and role in improving the online signing rate and promoting the incubation of CIFIT platform.

**Table 8:** Variance decomposition of Y_1_.

Period	S.E.	Y1	X4	X6
1	0.310843	100.000000	0.000000	0.000000
2	0.438929	95.770292	3.235723	0.993985
3	0.660536	65.572743	2.184326	32.242931
4	0.704772	65.188024	2.739865	32.072111
5	0.735739	67.619737	2.899229	29.481034
6	0.799377	69.100253	2.570230	28.329516
7	0.886019	65.637921	2.111524	32.250555
8	0.924179	66.381294	1.954633	31.664072
9	0.962972	68.164447	2.116027	29.719526
10	1.029932	68.450853	1.980420	29.568727
11	1.101562	67.530983	1.748621	30.720396
12	1.152137	68.276642	1.692196	30.031161
13	1.208908	69.368694	1.749991	28.881315
14	1.285572	69.417293	1.657397	28.925310
15	1.362485	69.230825	1.536867	29.232308
16	1.431090	69.803429	1.514458	28.682113
17	1.508378	70.388089	1.521512	28.090399
18	1.599284	70.410526	1.459322	28.130152
19	1.690791	70.451924	1.396596	28.151481
20	1.781367	70.833883	1.383948	27.782169
Cholesky ordering: Y1 X4 X6				

As shown in Table 9, the contribution of Y_1_ to X_4_ is increasing year by year, and the contribution is as high as 68.45528%. The contribution of X_6_ to X_4_ also shows an upward trend year by year, but the impact range is not as large as Y_1_ and the highest contribution is 25.34571%. This shows that the better the domestic business environment, the greater the attraction to X_4_. More and more business groups will participate in the CIFIT platform. The more business groups participate, the greater the impact on the contract-signing rate.

**Table 9:** Variance decomposition of X_4_.

Period	S.E.	Y1	X4	X6
1	0.310843	7.294194	92.705806	0.000000
2	0.438929	5.968136	86.218793	7.813070
3	0.660536	8.849093	82.184154	8.966754
4	0.704772	16.938220	73.269744	9.792036
5	0.735739	26.875023	58.932638	14.192339
6	0.799377	34.490993	47.054780	18.454228
7	0.886019	41.069409	39.461173	19.469418
8	0.924179	46.977395	33.021889	20.000716
9	0.962972	51.264537	27.161726	21.573737
10	1.029932	54.434319	22.750964	22.814717
11	1.101562	57.359997	19.545032	23.094970
12	1.152137	59.812557	16.838818	23.348625
13	1.208908	61.548843	14.478318	23.972839
14	1.285572	62.992076	12.592252	24.415672
15	1.362485	64.382395	11.086714	24.530891
16	1.431090	65.524198	9.784208	24.691594
17	1.508378	66.370698	8.645765	24.983537
18	1.599284	67.131994	7.696085	25.171922
19	1.690791	67.861390	6.899000	25.239610
20	1.781367	68.455275	6.199013	25.345712
Cholesky ordering: Y1 X4 X6				

In Table 10, the contribution of Y_1_ to X_6_ is increasing year by year, with a contribution of 40.00174%. The contribution of X_4_ to X_6_ is relatively flat, and the impact range is not as large as Y_1_. The highest contribution is 6.365912% of phase 3. Similarly, the better the domestic business environment, the higher the cooperation signing rate of the platform, the greater the attraction to X_6_ the more countries and regions take part in CIFIT. Y_1_ contributes more to X_4_ than X_6_. This shows that the cooperation and signing rate of CIFIT is more attractive to, X_4_ and industrial and commercial groups favor the project incubation of CIFIT platform. Therefore, the effect of Y_1_ on X_4_ and X_6_ is significant. This also provides theoretical support for us to take various measures to improve the “Online + Offline” mode and facilitate a sustainable development of CIFIT in investment, trade, and project incubation.

**Table 10:** Variance decomposition of X_6_.

Period	S.E.	Y1	X4	X6
1	0.310843	20.375836	2.123086	77.501077
2	0.438929	16.861601	5.150779	77.987621
3	0.660536	20.298197	6.365912	73.335891
4	0.704772	22.710791	6.023095	71.266114
5	0.735739	22.293347	6.121465	71.585188
6	0.799377	21.991342	6.031304	71.977354
7	0.886019	23.773661	6.228288	69.998051
8	0.924179	24.799465	5.738091	69.462445
9	0.962972	25.225710	5.638480	69.135810
10	1.029932	25.827747	5.716818	68.455435
11	1.101562	27.569414	5.684192	66.746395
12	1.152137	28.623317	5.355440	66.021243
13	1.208908	29.537032	5.226514	65.236454
14	1.285572	30.810098	5.248141	63.941761
15	1.362485	32.550556	5.109527	62.339917
16	1.431090	33.819846	4.851787	61.328367
17	1.508378	35.143696	4.718495	60.137809
18	1.599284	36.809072	4.658185	58.532743
19	1.690791	38.542697	4.479649	56.977655
20	1.781367	40.001745	4.260068	55.738187
Cholesky ordering: Y1 X4 X6				

## 5. Conclusions and policy recommendation

From the empirical analysis results, it can be concluded that the average comprehensive resource allocation efficiency of CIFIT is 0.83, showing phased characteristics in 20 years. There are both differences and similarities in each stage, reflecting the characteristics of high PTE. There is also a mismatch between the phased resource allocation scale and the optimal scale, resulting in low allocation efficiency and low comprehensive efficiency. However, the overall resource allocation efficiency of CIFIT is well. Although it does not fully reach DEA efficiency, the overall trend shows increasing efficiency of scale. Through analysis, this study finds several main factors affecting its resource allocation efficiency.

First, the utilization of exhibition scale and the innovation of booth design affect the efficiency of resource allocation. From several site expansions, it can be seen that the market demand and the scale of the platform show increasing benefits, but in the transition year of capacity expansion, it is easy to have ineffective comprehensive efficiency. The main reason is that the scale benefits are also ineffective. Redundant investment occurs, but the scale benefit increases, mainly due to the low scale utilization rate of the site. Especially during the pandemic, there is a great deal of redundancy in investment. The utilization rate of site scale is closely related to the innovation of booth design. If the site scale is used as a necessary input, the booth design innovation is soft power. Through the corresponding systematic, innovative design, the site utilization can be improved accordingly, and the hard power can be improved through soft power.

Second, the project strength of participating enterprises and the signing rate of overseas customers affect the efficiency of resource allocation. CIFIT is an international investment and trade stage and an international project incubation platform. The level of participating enterprises and the scale of declared projects determine the signing rate of projects to a certain extent, while the signing rate of overseas customers directly affects the external funds used by the platform. These two factors not only have a direct impact on output indicators but also cross-affect each other. Under the background of China’s Belt and Road Initiative, exchanges between China and other countries along the Belt and Road coastal areas have been deepening. It needs to improve the quality of projects to improve efficiency and achieve high-quality development step by step.

At the same time, the study uses a VAR model to perform empirical analysis, and the following conclusions are drawn: the number of industrial and commercial groups and participating countries and regions are Granger causality for Y_1_; the effect of Y_1_ on X_4_ and X_6_ is significant; the “Online + Offline” mode should focus on these two aspects.

Based on the above conclusions, this paper puts forward relevant policy suggestions in the post pandemic era:

Firstly, strengthen the integration of the “Online + Offline” mode to improve resource utilization efficiency. Especially during the pandemic, many offline problems can be solved online, and the effects that cannot be achieved offline can be filled through online digitization and scale. This is not only to solve the problem during the pandemic but also to explore “Online + Offline” after the pandemic is over. The pandemic situation brings both challenges and opportunities. The combination of “Online + Offline” is also the best transition from traditional international exhibitions to online. On the one hand, offline is an important traditional mode for CIFIT from the perspective of traditional efficiency and effect, it still needs the offline mode, reflecting more humanistic factors. Significant project investments also need face-to-face communication, exchange, and sustainable trust. On the other hand, the online mode is cost-effective and fast. The “cloud” is the superposition of 3D space. The value of the exhibition is to save transaction costs. The SE of the site cannot be matched offline, and “Cloud Exhibition” can be used to make up for the SE of the site. This is an innovation of the transaction mode. Cloud exhibition borrows the platform’s value, saves transaction costs, and provides a new path to improve the utilization of offline resources.

Second, the optimization of the business environment of online exhibitions and an improved design of cooperation system to broaden the path of online exhibition further. The exhibition is a mode to save transaction costs. Cloud exhibition is the best transaction mode during the pandemic, and it is also a new path to expand the high-quality development of “Online + Offline” in the future. The innovative development space of “Cloud Exhibition” can be explored from four aspects. First, the problem of brand and intellectual property rights, and how much transaction costs the exhibition saves; that is, the intangible assets of the exhibition. Second, technological innovation and strong technical security must be implemented to prevent hacker intrusion and the spread of malicious and inappropriate software. Third, continuous iterative innovation of technology must be ensured to prevent the problem of login collapse when the platform is at its peak and tens of thousands of merchants are online. Finally, an automatic emergency mechanism must be established to prevent major political, economic, and technological risks. Therefore, through the combination of online and offline modes, the offline efficiency can be better, giving CIFIT a new path to realize the high-quality development of the international convention and exhibition platform.

Third, the continuous annual exhibition and strengthen the incubation function of the platform must be restored. From the perspective of increasing returns to scale, CIFIT can be held continuously every year to achieve comprehensive optimal efficiency. On the contrary, holding them in alternating years will have the lag effect on optimal comprehensive efficiency. Further, it can restore the annual mechanism, strengthen the incubation function of the platform, drive the establishment of incubation base and industrial park through the project landing, and promote the in-depth cooperation between the project and financial capital. Finally, according to the exhibition’s space-time characteristics, industrial characteristics, and cultural attributes, it can be divided into online and offline exhibitions, changing the traditional mode of special and single industry exhibitions. Professional exhibitions can be mainly offline, and some overseas exhibitions can be mainly online. This transforms the CIFIT into a 3D exhibition, becoming a kind of “Composite International Exhibition” combining domestic and overseas, online and offline, and investment and trade.

Overall, the resource allocation efficiency of CIFIT is good, which promotes the regional economic growth in Xiamen to a large extent. This is a solid foundation for Xiamen to become an international city. Also, under the background of the Belt and Road Initiative, the core area of the Maritime Silk Road provides Xiamen with the support of global city development. Xiamen’s upgrading of soft and hard facilities is not only to accelerate the city’s future development but also to gradually consolidate the core area of the Maritime Silk Road and become a regional engine of the “global city” driving the world economy. Through the annual incubation and transformation of the CIFIT platform, Xiamen has gradually formed a unique solid foundation, good business environment, and hard strength for investment services throughout the country and around the world.

## Supporting information

S1 Fig Resource allocation efficiency of CIFIT 2001–2019 under BCC mode. (DOCX)

S2 Fig Comparison of resource allocation efficiency of CIFIT between 2001–2019 and 2001–2020. (DOCX)

S3 Fig Stability test of VAR model. (DOCX)

S4 Fig VAR pulse function diagram. (DOCX)

S1 Text (DOCX)

S2 Text (DOCX)

S1 File (DOCX)

S2 File (DOCX)
