# Supplementary figures and images for: Research on the transformation from international exhibition to “cloud” exhibition in the post COVID-19 era: A case study of China International Fair for Investment & Trade
Source: PLoS One. 2022 Apr 28;17(4):e0267455. doi: 10.1371/journal.pone.0267455 (PMC9049316; doi:10.1371/journal.pone.0267455)

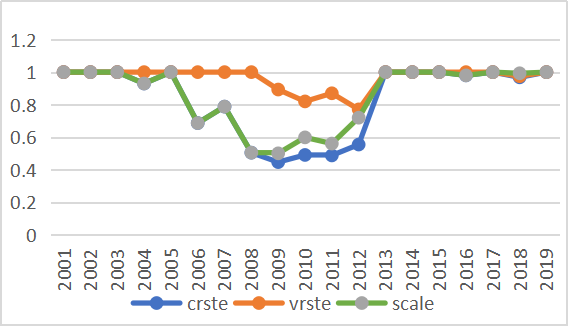

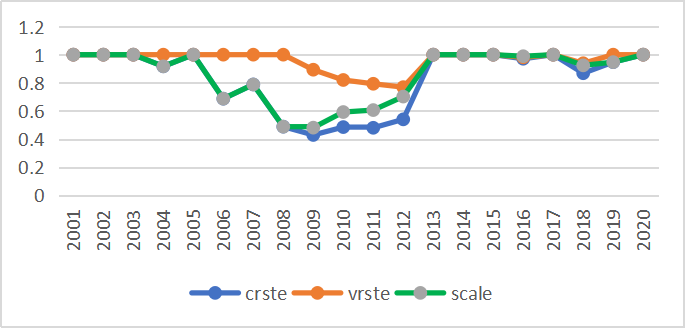

Supplement: S2 Fig — (DOCX) [file pone.0267455.s002.docx]

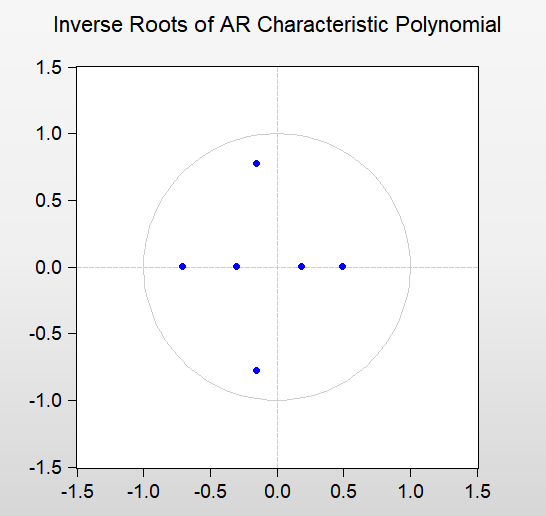

Supplement: S3 Fig — (DOCX) [file pone.0267455.s003.docx]

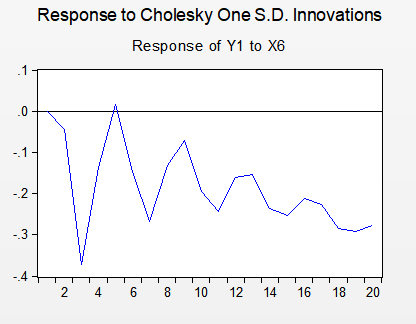

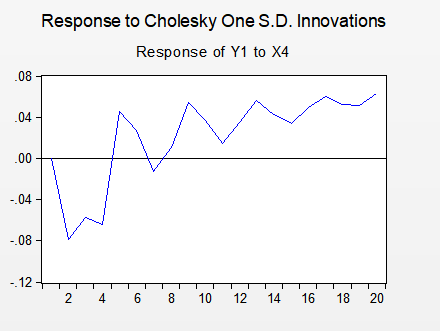

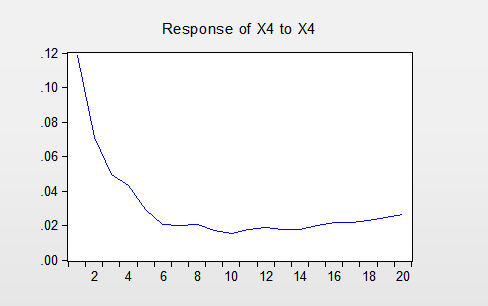

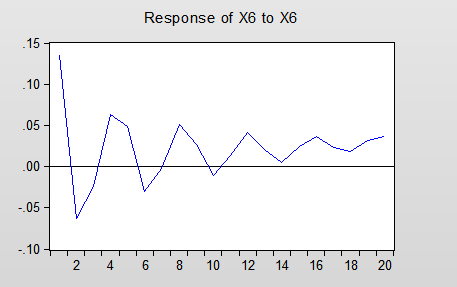

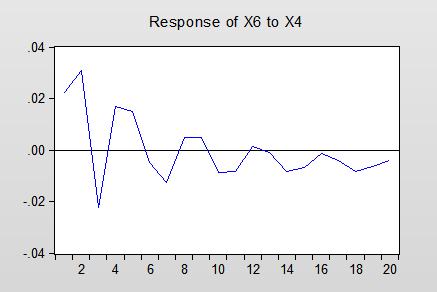

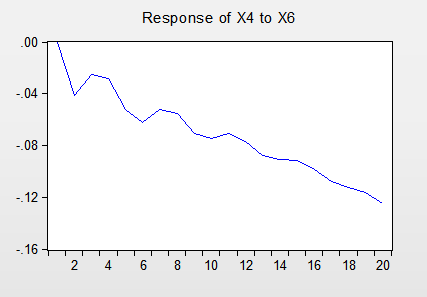

Supplement: S4 Fig — (DOCX) [file pone.0267455.s004.docx]
