# Supplementary material for: Research on the transformation from international exhibition to “cloud” exhibition in the post COVID-19 era: A case study of China International Fair for Investment & Trade
Source: PLoS One. 2022 Apr 28;17(4):e0267455. doi: 10.1371/journal.pone.0267455 (PMC9049316; doi:10.1371/journal.pone.0267455)
Supplement: S1 Text — (DOCX) [file pone.0267455.s005.docx]

FIRM BY FIRM RESULTS:

Results for firm: 1

Technical efficiency = 1.000

Scale efficiency = 1.000 (crs)

PROJECTION SUMMARY:

variable original radial slack projected

value movement movement value

output 1 1027.000 0.000 0.000 1027.000

output 2 47.980 0.000 0.000 47.980

input 1 28000.000 0.000 0.000 28000.000

input 2 2500.000 0.000 0.000 2500.000

input 3 500.000 0.000 0.000 500.000

input 4 117.000 0.000 0.000 117.000

input 5 1100.000 0.000 0.000 1100.000

input 6 97.000 0.000 0.000 97.000

LISTING OF PEERS:

peer lambda weight

1 1.000

Results for firm: 2

Technical efficiency = 1.000

Scale efficiency = 1.000 (crs)

PROJECTION SUMMARY:

variable original radial slack projected

value movement movement value

output 1 1151.000 0.000 0.000 1151.000

output 2 51.910 0.000 0.000 51.910

input 1 28000.000 0.000 0.000 28000.000

input 2 2500.000 0.000 0.000 2500.000

input 3 460.000 0.000 0.000 460.000

input 4 141.000 0.000 0.000 141.000

input 5 1032.000 0.000 0.000 1032.000

input 6 96.000 0.000 0.000 96.000

LISTING OF PEERS:

peer lambda weight

2 1.000

Results for firm: 3

Technical efficiency = 1.000

Scale efficiency = 1.000 (crs)

PROJECTION SUMMARY:

variable original radial slack projected

value movement movement value

output 1 1259.000 0.000 0.000 1259.000

output 2 66.520 0.000 0.000 66.520

input 1 28000.000 0.000 0.000 28000.000

input 2 2500.000 0.000 0.000 2500.000

input 3 421.000 0.000 0.000 421.000

input 4 240.000 0.000 0.000 240.000

input 5 1200.000 0.000 0.000 1200.000

input 6 102.000 0.000 0.000 102.000

LISTING OF PEERS:

peer lambda weight

3 1.000

Results for firm: 4

Technical efficiency = 1.000

Scale efficiency = 0.930 (irs)

PROJECTION SUMMARY:

variable original radial slack projected

value movement movement value

output 1 1110.000 0.000 6.749 1116.749

output 2 81.220 0.000 0.000 81.220

input 1 33000.000 0.000 -2974.186 30025.814

input 2 2500.000 0.000 0.000 2500.000

input 3 520.000 0.000 -56.298 463.702

input 4 300.000 0.000 -69.321 230.679

input 5 1296.000 0.000 -109.251 1186.749

input 6 118.000 0.000 -9.947 108.053

LISTING OF PEERS:

peer lambda weight

2 0.544

5 0.405

3 0.051

Results for firm: 5

Technical efficiency = 1.000

Scale efficiency = 1.000 (crs)

PROJECTION SUMMARY:

variable original radial slack projected

value movement movement value

output 1 1053.000 0.000 0.000 1053.000

output 2 122.430 0.000 0.000 122.430

input 1 33000.000 0.000 0.000 33000.000

input 2 2500.000 0.000 0.000 2500.000

input 3 474.000 0.000 0.000 474.000

input 4 350.000 0.000 0.000 350.000

input 5 1393.000 0.000 0.000 1393.000

input 6 125.000 0.000 0.000 125.000

LISTING OF PEERS:

peer lambda weight

5 1.000

Results for firm: 6

Technical efficiency = 1.000

Scale efficiency = 0.687 (irs)

PROJECTION SUMMARY:

variable original radial slack projected

value movement movement value

output 1 752.000 0.000 471.445 1223.445

output 2 76.170 0.000 0.000 76.170

input 1 33000.000 0.000 -4137.006 28862.994

input 2 2500.000 0.000 0.000 2500.000

input 3 731.000 0.000 -300.852 430.148

input 4 383.000 0.000 -124.014 258.986

input 5 1389.000 0.000 -155.688 1233.312

input 6 113.000 0.000 -7.030 105.970

LISTING OF PEERS:

peer lambda weight

3 0.827

5 0.173

Results for firm: 7

Technical efficiency = 1.000

Scale efficiency = 0.788 (irs)

PROJECTION SUMMARY:

variable original radial slack projected

value movement movement value

output 1 669.000 0.000 480.644 1149.644

output 2 96.200 0.000 0.000 96.200

input 1 33000.000 0.000 -2345.734 30654.266

input 2 2500.000 0.000 0.000 2500.000

input 3 1053.000 0.000 -603.865 449.135

input 4 414.000 0.000 -115.606 298.394

input 5 1385.000 0.000 -82.545 1302.455

input 6 119.000 0.000 -4.790 114.210

LISTING OF PEERS:

peer lambda weight

5 0.531

3 0.469

Results for firm: 8

Technical efficiency = 1.000

Scale efficiency = 0.505 (irs)

PROJECTION SUMMARY:

variable original radial slack projected

value movement movement value

output 1 517.000 0.000 609.845 1126.845

output 2 80.990 0.000 0.000 80.990

input 1 52000.000 0.000 -22069.425 29930.575

input 2 2500.000 0.000 0.000 2500.000

input 3 1246.000 0.000 -785.536 460.464

input 4 445.000 0.000 -210.758 234.242

input 5 1392.000 0.000 -199.326 1192.674

input 6 126.000 0.000 -18.042 107.958

LISTING OF PEERS:

peer lambda weight

5 0.386

2 0.487

3 0.127

Results for firm: 9

Technical efficiency = 0.893

Scale efficiency = 0.501 (irs)

PROJECTION SUMMARY:

variable original radial slack projected

value movement movement value

output 1 515.000 0.000 646.391 1161.391

output 2 75.300 0.000 0.000 75.300

input 1 60000.000 -6428.571 -24259.360 29312.069

input 2 2800.000 -300.000 0.000 2500.000

input 3 1157.000 -123.964 -582.401 450.635

input 4 492.000 -52.714 -210.342 228.943

input 5 1400.000 -150.000 -67.101 1182.899

input 6 125.000 -13.393 -5.991 105.616

LISTING OF PEERS:

peer lambda weight

5 0.262

2 0.403

3 0.334

Results for firm: 10

Technical efficiency = 0.820

Scale efficiency = 0.599 (irs)

PROJECTION SUMMARY:

variable original radial slack projected

value movement movement value

output 1 484.000 0.000 676.082 1160.082

output 2 99.600 0.000 0.000 99.600

input 1 65000.000 -11712.955 -17351.825 35935.220

input 2 3214.000 -579.161 0.000 2634.839

input 3 1378.000 -248.315 -347.368 782.317

input 4 627.000 -112.985 -266.109 247.906

input 5 1439.000 -259.307 0.000 1179.693

input 6 144.000 -25.949 -12.757 105.294

LISTING OF PEERS:

peer lambda weight

15 0.090

5 0.293

2 0.618

Results for firm: 11

Technical efficiency = 0.870

Scale efficiency = 0.561 (irs)

PROJECTION SUMMARY:

variable original radial slack projected

value movement movement value

output 1 339.000 0.000 874.180 1213.180

output 2 104.600 0.000 0.000 104.600

input 1 65000.000 -8463.228 -9050.170 47486.602

input 2 4000.000 -520.814 -359.158 3120.028

input 3 1600.000 -208.326 -127.409 1264.265

input 4 636.000 -82.809 -311.923 241.267

input 5 1478.000 -192.441 -206.083 1079.476

input 6 112.000 -14.583 0.000 97.417

LISTING OF PEERS:

peer lambda weight

2 0.823

16 0.177

Results for firm: 12

Technical efficiency = 0.769

Scale efficiency = 0.719 (irs)

PROJECTION SUMMARY:

variable original radial slack projected

value movement movement value

output 1 355.000 0.000 937.782 1292.782

output 2 154.200 0.000 0.000 154.200

input 1 100000.000 -23087.960 -23247.187 53664.853

input 2 4000.000 -923.518 0.000 3076.482

input 3 2200.000 -507.935 -37.802 1654.263

input 4 650.000 -150.072 -184.040 315.888

input 5 1500.000 -346.319 0.000 1153.681

input 6 132.000 -30.476 -2.352 99.172

LISTING OF PEERS:

peer lambda weight

2 0.663

15 0.265

17 0.072

Results for firm: 13

Technical efficiency = 1.000

Scale efficiency = 1.000 (crs)

PROJECTION SUMMARY:

variable original radial slack projected

value movement movement value

output 1 1386.000 0.000 0.000 1386.000

output 2 321.600 0.000 0.000 321.600

input 1 100000.000 0.000 0.000 100000.000

input 2 4000.000 0.000 0.000 4000.000

input 3 2870.000 0.000 0.000 2870.000

input 4 556.000 0.000 0.000 556.000

input 5 1500.000 0.000 0.000 1500.000

input 6 118.000 0.000 0.000 118.000

LISTING OF PEERS:

peer lambda weight

13 1.000

Results for firm: 14

Technical efficiency = 1.000

Scale efficiency = 1.000 (crs)

PROJECTION SUMMARY:

variable original radial slack projected

value movement movement value

output 1 1455.000 0.000 0.000 1455.000

output 2 336.400 0.000 0.000 336.400

input 1 100000.000 0.000 0.000 100000.000

input 2 4000.000 0.000 0.000 4000.000

input 3 3000.000 0.000 0.000 3000.000

input 4 603.000 0.000 0.000 603.000

input 5 1500.000 0.000 0.000 1500.000

input 6 126.000 0.000 0.000 126.000

LISTING OF PEERS:

peer lambda weight

14 1.000

Results for firm: 15

Technical efficiency = 1.000

Scale efficiency = 1.000 (crs)

PROJECTION SUMMARY:

variable original radial slack projected

value movement movement value

output 1 1571.000 0.000 0.000 1571.000

output 2 352.900 0.000 0.000 352.900

input 1 100000.000 0.000 0.000 100000.000

input 2 4000.000 0.000 0.000 4000.000

input 3 4000.000 0.000 0.000 4000.000

input 4 650.000 0.000 0.000 650.000

input 5 1500.000 0.000 0.000 1500.000

input 6 105.000 0.000 0.000 105.000

LISTING OF PEERS:

peer lambda weight

15 1.000

Results for firm: 16

Technical efficiency = 1.000

Scale efficiency = 0.980 (irs)

PROJECTION SUMMARY:

variable original radial slack projected

value movement movement value

output 1 1502.000 0.000 0.000 1502.000

output 2 349.340 0.000 0.000 349.340

input 1 138000.000 0.000 0.000 138000.000

input 2 6000.000 0.000 0.000 6000.000

input 3 5000.000 0.000 0.000 5000.000

input 4 707.000 0.000 0.000 707.000

input 5 1300.000 0.000 0.000 1300.000

input 6 104.000 0.000 0.000 104.000

LISTING OF PEERS:

peer lambda weight

16 1.000

Results for firm: 17

Technical efficiency = 1.000

Scale efficiency = 1.000 (crs)

PROJECTION SUMMARY:

variable original radial slack projected

value movement movement value

output 1 1577.000 0.000 0.000 1577.000

output 2 366.730 0.000 0.000 366.730

input 1 120000.000 0.000 0.000 120000.000

input 2 5000.000 0.000 0.000 5000.000

input 3 4040.000 0.000 0.000 4040.000

input 4 700.000 0.000 0.000 700.000

input 5 1000.000 0.000 0.000 1000.000

input 6 107.000 0.000 0.000 107.000

LISTING OF PEERS:

peer lambda weight

17 1.000

Results for firm: 18

Technical efficiency = 0.973

Scale efficiency = 0.994 (irs)

PROJECTION SUMMARY:

variable original radial slack projected

value movement movement value

output 1 1982.000 0.000 0.000 1982.000

output 2 365.200 0.000 6.275 371.475

input 1 130000.000 -3491.964 -1615.651 124892.385

input 2 6000.000 -161.168 -206.201 5632.631

input 3 5000.000 -134.306 -1638.494 3227.200

input 4 1005.000 -26.996 -46.159 931.846

input 5 1500.000 -40.292 0.000 1459.708

input 6 128.000 -3.438 0.000 124.562

LISTING OF PEERS:

peer lambda weight

15 0.143

19 0.776

17 0.081

Results for firm: 19

Technical efficiency = 1.000

Scale efficiency = 1.000 (crs)

PROJECTION SUMMARY:

variable original radial slack projected

value movement movement value

output 1 2100.000 0.000 0.000 2100.000

output 2 375.400 0.000 0.000 375.400

input 1 130000.000 0.000 0.000 130000.000

input 2 6000.000 0.000 0.000 6000.000

input 3 3000.000 0.000 0.000 3000.000

input 4 1008.000 0.000 0.000 1008.000

input 5 1500.000 0.000 0.000 1500.000

input 6 130.000 0.000 0.000 130.000

LISTING OF PEERS:

peer lambda weight

19 1.000
