# Supplementary material for: Research on the transformation from international exhibition to “cloud” exhibition in the post COVID-19 era: A case study of China International Fair for Investment & Trade
Source: PLoS One. 2022 Apr 28;17(4):e0267455. doi: 10.1371/journal.pone.0267455 (PMC9049316; doi:10.1371/journal.pone.0267455)
Supplement: S2 File — (DOCX) [file pone.0267455.s008.docx]

Results from DEAP Version 2.1

Instruction file = 98-2-20.ins

Data file = 98-2-20.dta

Input orientated DEA

Scale assumption: VRS

Slacks calculated using multi-stage method

EFFICIENCY SUMMARY:

firm crste vrste scale

1 1.000 1.000 1.000 -

2 1.000 1.000 1.000 -

3 1.000 1.000 1.000 -

4 0.917 1.000 0.917 irs

5 1.000 1.000 1.000 -

6 0.687 1.000 0.687 irs

7 0.788 1.000 0.788 irs

8 0.488 1.000 0.488 irs

9 0.429 0.893 0.481 irs

10 0.485 0.820 0.592 irs

11 0.480 0.793 0.606 irs

12 0.540 0.769 0.702 irs

13 1.000 1.000 1.000 -

14 1.000 1.000 1.000 -

15 1.000 1.000 1.000 -

16 0.971 0.981 0.989 irs

17 1.000 1.000 1.000 -

18 0.868 0.939 0.924 drs

19 0.947 1.000 0.947 drs

20 1.000 1.000 1.000 -

mean 0.830 0.960 0.856

Note: crste = technical efficiency from CRS DEA

vrste = technical efficiency from VRS DEA

scale = scale efficiency = crste/vrste

Note also that all subsequent tables refer to VRS results

SUMMARY OF OUTPUT SLACKS:

firm output: 1 2

1 0.000 0.000

2 0.000 0.000

3 0.000 0.000

4 6.749 0.000

5 0.000 0.000

6 471.445 0.000

7 480.644 0.000

8 609.845 0.000

9 646.391 0.000

10 676.082 0.000

11 1119.979 15.819

12 980.299 0.000

13 0.000 0.000

14 0.000 0.000

15 0.000 0.000

16 135.414 0.000

17 0.000 0.000

18 0.000 0.000

19 0.000 0.000

20 0.000 0.000

mean 256.342 0.791

SUMMARY OF INPUT SLACKS:

firm input: 1 2 3 4 5 6

1 0.000 0.000 0.000 0.000 0.000 0.000

2 0.000 0.000 0.000 0.000 0.000 0.000

3 0.000 0.000 0.000 0.000 0.000 0.000

4 2974.186 0.000 56.298 69.321 109.251 9.947

5 0.000 0.000 0.000 0.000 0.000 0.000

6 4137.006 0.000 300.852 124.014 155.688 7.030

7 2345.734 0.000 603.865 115.606 82.545 4.790

8 22069.425 0.000 785.536 210.758 199.326 18.042

9 24259.360 0.000 582.401 210.342 67.101 5.991

10 17351.825 0.000 347.368 266.109 0.000 12.757

11 1534.794 0.000 395.258 127.974 228.343 0.000

12 23289.219 0.000 104.603 162.227 0.000 4.204

13 0.000 0.000 0.000 0.000 0.000 0.000

14 0.000 0.000 0.000 0.000 0.000 0.000

15 0.000 0.000 0.000 0.000 0.000 0.000

16 29514.191 1522.605 1122.922 0.000 0.000 0.000

17 0.000 0.000 0.000 0.000 0.000 0.000

18 0.000 157.380 1515.463 22.225 0.000 1.128

19 0.000 0.000 0.000 0.000 0.000 0.000

20 0.000 0.000 0.000 0.000 0.000 0.000

mean 6373.787 83.999 290.728 65.429 42.113 3.195

SUMMARY OF PEERS:

firm peers:

1 1

2 2

3 3

4 2 5 3

5 5

6 5 3

7 5 3

8 5 2 3

9 5 2 3

10 2 5 15

11 20 2

12 2 15 20

13 13

14 14

15 15

16 20 17 15 2

17 17

18 19 20 15 17

19 19

20 20

SUMMARY OF PEER WEIGHTS:

(in same order as above)

firm peer weights:

1 1.000

2 1.000

3 1.000

4 0.544 0.405 0.051

5 1.000

6 0.173 0.827

7 0.531 0.469

8 0.386 0.487 0.127

9 0.262 0.403 0.334

10 0.618 0.293 0.090

11 0.268 0.732

12 0.652 0.295 0.052

13 1.000

14 1.000

15 1.000

16 0.095 0.287 0.608 0.011

17 1.000

18 0.668 0.078 0.194 0.059

19 1.000

20 1.000

PEER COUNT SUMMARY:

(i.e., no. times each firm is a peer for another)

firm peer count:

1 0

2 7

3 5

4 0

5 6

6 0

7 0

8 0

9 0

10 0

11 0

12 0

13 0

14 0

15 4

16 0

17 2

18 0

19 1

20 4

SUMMARY OF OUTPUT TARGETS:

firm output: 1 2

1 1027.000 47.980

2 1151.000 51.910

3 1259.000 66.520

4 1116.749 81.220

5 1053.000 122.430

6 1223.445 76.170

7 1149.644 96.200

8 1126.845 80.990

9 1161.391 75.300

10 1160.082 99.600

11 1458.979 120.419

12 1335.299 154.200

13 1386.000 321.600

14 1455.000 336.400

15 1571.000 352.900

16 1637.414 349.340

17 1577.000 366.730

18 1982.000 365.200

19 2100.000 375.400

20 2300.000 307.500

SUMMARY OF INPUT TARGETS:

firm input: 1 2 3 4 5 6

1 28000.000 2500.000 500.000 117.000 1100.000 97.000

2 28000.000 2500.000 460.000 141.000 1032.000 96.000

3 28000.000 2500.000 421.000 240.000 1200.000 102.000

4 30025.814 2500.000 463.702 230.679 1186.749 108.053

5 33000.000 2500.000 474.000 350.000 1393.000 125.000

6 28862.994 2500.000 430.148 258.986 1233.312 105.970

7 30654.266 2500.000 449.135 298.394 1302.455 114.210

8 29930.575 2500.000 460.464 234.242 1192.674 107.958

9 29312.069 2500.000 450.635 228.943 1182.899 105.616

10 35935.220 2634.839 782.317 247.906 1179.693 105.294

11 49979.381 3170.103 872.784 376.072 943.010 88.763

12 53563.113 3074.093 1586.148 337.313 1152.785 97.241

13 100000.000 4000.000 2870.000 556.000 1500.000 118.000

14 100000.000 4000.000 3000.000 603.000 1500.000 126.000

15 100000.000 4000.000 4000.000 650.000 1500.000 105.000

16 105912.572 4365.515 3783.845 693.817 1275.759 102.061

17 120000.000 5000.000 4040.000 700.000 1000.000 107.000

18 122014.398 5474.054 3177.399 921.041 1407.858 119.009

19 130000.000 6000.000 3000.000 1008.000 1500.000 130.000

20 110000.000 5000.000 2000.000 1018.000 700.000 69.000

FIRM BY FIRM RESULTS:

Results for firm: 1

Technical efficiency = 1.000

Scale efficiency = 1.000 (crs)

PROJECTION SUMMARY:

variable original radial slack projected

value movement movement value

output 1 1027.000 0.000 0.000 1027.000

output 2 47.980 0.000 0.000 47.980

input 1 28000.000 0.000 0.000 28000.000

input 2 2500.000 0.000 0.000 2500.000

input 3 500.000 0.000 0.000 500.000

input 4 117.000 0.000 0.000 117.000

input 5 1100.000 0.000 0.000 1100.000

input 6 97.000 0.000 0.000 97.000

LISTING OF PEERS:

peer lambda weight

1 1.000

Results for firm: 2

Technical efficiency = 1.000

Scale efficiency = 1.000 (crs)

PROJECTION SUMMARY:

variable original radial slack projected

value movement movement value

output 1 1151.000 0.000 0.000 1151.000

output 2 51.910 0.000 0.000 51.910

input 1 28000.000 0.000 0.000 28000.000

input 2 2500.000 0.000 0.000 2500.000

input 3 460.000 0.000 0.000 460.000

input 4 141.000 0.000 0.000 141.000

input 5 1032.000 0.000 0.000 1032.000

input 6 96.000 0.000 0.000 96.000

LISTING OF PEERS:

peer lambda weight

2 1.000

Results for firm: 3

Technical efficiency = 1.000

Scale efficiency = 1.000 (crs)

PROJECTION SUMMARY:

variable original radial slack projected

value movement movement value

output 1 1259.000 0.000 0.000 1259.000

output 2 66.520 0.000 0.000 66.520

input 1 28000.000 0.000 0.000 28000.000

input 2 2500.000 0.000 0.000 2500.000

input 3 421.000 0.000 0.000 421.000

input 4 240.000 0.000 0.000 240.000

input 5 1200.000 0.000 0.000 1200.000

input 6 102.000 0.000 0.000 102.000

LISTING OF PEERS:

peer lambda weight

3 1.000

Results for firm: 4

Technical efficiency = 1.000

Scale efficiency = 0.917 (irs)

PROJECTION SUMMARY:

variable original radial slack projected

value movement movement value

output 1 1110.000 0.000 6.749 1116.749

output 2 81.220 0.000 0.000 81.220

input 1 33000.000 0.000 -2974.186 30025.814

input 2 2500.000 0.000 0.000 2500.000

input 3 520.000 0.000 -56.298 463.702

input 4 300.000 0.000 -69.321 230.679

input 5 1296.000 0.000 -109.251 1186.749

input 6 118.000 0.000 -9.947 108.053

LISTING OF PEERS:

peer lambda weight

2 0.544

5 0.405

3 0.051

Results for firm: 5

Technical efficiency = 1.000

Scale efficiency = 1.000 (crs)

PROJECTION SUMMARY:

variable original radial slack projected

value movement movement value

output 1 1053.000 0.000 0.000 1053.000

output 2 122.430 0.000 0.000 122.430

input 1 33000.000 0.000 0.000 33000.000

input 2 2500.000 0.000 0.000 2500.000

input 3 474.000 0.000 0.000 474.000

input 4 350.000 0.000 0.000 350.000

input 5 1393.000 0.000 0.000 1393.000

input 6 125.000 0.000 0.000 125.000

LISTING OF PEERS:

peer lambda weight

5 1.000

Results for firm: 6

Technical efficiency = 1.000

Scale efficiency = 0.687 (irs)

PROJECTION SUMMARY:

variable original radial slack projected

value movement movement value

output 1 752.000 0.000 471.445 1223.445

output 2 76.170 0.000 0.000 76.170

input 1 33000.000 0.000 -4137.006 28862.994

input 2 2500.000 0.000 0.000 2500.000

input 3 731.000 0.000 -300.852 430.148

input 4 383.000 0.000 -124.014 258.986

input 5 1389.000 0.000 -155.688 1233.312

input 6 113.000 0.000 -7.030 105.970

LISTING OF PEERS:

peer lambda weight

5 0.173

3 0.827

Results for firm: 7

Technical efficiency = 1.000

Scale efficiency = 0.788 (irs)

PROJECTION SUMMARY:

variable original radial slack projected

value movement movement value

output 1 669.000 0.000 480.644 1149.644

output 2 96.200 0.000 0.000 96.200

input 1 33000.000 0.000 -2345.734 30654.266

input 2 2500.000 0.000 0.000 2500.000

input 3 1053.000 0.000 -603.865 449.135

input 4 414.000 0.000 -115.606 298.394

input 5 1385.000 0.000 -82.545 1302.455

input 6 119.000 0.000 -4.790 114.210

LISTING OF PEERS:

peer lambda weight

5 0.531

3 0.469

Results for firm: 8

Technical efficiency = 1.000

Scale efficiency = 0.488 (irs)

PROJECTION SUMMARY:

variable original radial slack projected

value movement movement value

output 1 517.000 0.000 609.845 1126.845

output 2 80.990 0.000 0.000 80.990

input 1 52000.000 0.000 -22069.425 29930.575

input 2 2500.000 0.000 0.000 2500.000

input 3 1246.000 0.000 -785.536 460.464

input 4 445.000 0.000 -210.758 234.242

input 5 1392.000 0.000 -199.326 1192.674

input 6 126.000 0.000 -18.042 107.958

LISTING OF PEERS:

peer lambda weight

5 0.386

2 0.487

3 0.127

Results for firm: 9

Technical efficiency = 0.893

Scale efficiency = 0.481 (irs)

PROJECTION SUMMARY:

variable original radial slack projected

value movement movement value

output 1 515.000 0.000 646.391 1161.391

output 2 75.300 0.000 0.000 75.300

input 1 60000.000 -6428.571 -24259.360 29312.069

input 2 2800.000 -300.000 0.000 2500.000

input 3 1157.000 -123.964 -582.401 450.635

input 4 492.000 -52.714 -210.342 228.943

input 5 1400.000 -150.000 -67.101 1182.899

input 6 125.000 -13.393 -5.991 105.616

LISTING OF PEERS:

peer lambda weight

5 0.262

2 0.403

3 0.334

Results for firm: 10

Technical efficiency = 0.820

Scale efficiency = 0.592 (irs)

PROJECTION SUMMARY:

variable original radial slack projected

value movement movement value

output 1 484.000 0.000 676.082 1160.082

output 2 99.600 0.000 0.000 99.600

input 1 65000.000 -11712.955 -17351.825 35935.220

input 2 3214.000 -579.161 0.000 2634.839

input 3 1378.000 -248.315 -347.368 782.317

input 4 627.000 -112.985 -266.109 247.906

input 5 1439.000 -259.307 0.000 1179.693

input 6 144.000 -25.949 -12.757 105.294

LISTING OF PEERS:

peer lambda weight

2 0.618

5 0.293

15 0.090

Results for firm: 11

Technical efficiency = 0.793

Scale efficiency = 0.606 (irs)

PROJECTION SUMMARY:

variable original radial slack projected

value movement movement value

output 1 339.000 0.000 1119.979 1458.979

output 2 104.600 0.000 15.819 120.419

input 1 65000.000 -13485.825 -1534.794 49979.381

input 2 4000.000 -829.897 0.000 3170.103

input 3 1600.000 -331.959 -395.258 872.784

input 4 636.000 -131.954 -127.974 376.072

input 5 1478.000 -306.647 -228.343 943.010

input 6 112.000 -23.237 0.000 88.763

LISTING OF PEERS:

peer lambda weight

20 0.268

2 0.732

Results for firm: 12

Technical efficiency = 0.769

Scale efficiency = 0.702 (irs)

PROJECTION SUMMARY:

variable original radial slack projected

value movement movement value

output 1 355.000 0.000 980.299 1335.299

output 2 154.200 0.000 0.000 154.200

input 1 100000.000 -23147.668 -23289.219 53563.113

input 2 4000.000 -925.907 0.000 3074.093

input 3 2200.000 -509.249 -104.603 1586.148

input 4 650.000 -150.460 -162.227 337.313

input 5 1500.000 -347.215 0.000 1152.785

input 6 132.000 -30.555 -4.204 97.241

LISTING OF PEERS:

peer lambda weight

2 0.652

15 0.295

20 0.052

Results for firm: 13

Technical efficiency = 1.000

Scale efficiency = 1.000 (crs)

PROJECTION SUMMARY:

variable original radial slack projected

value movement movement value

output 1 1386.000 0.000 0.000 1386.000

output 2 321.600 0.000 0.000 321.600

input 1 100000.000 0.000 0.000 100000.000

input 2 4000.000 0.000 0.000 4000.000

input 3 2870.000 0.000 0.000 2870.000

input 4 556.000 0.000 0.000 556.000

input 5 1500.000 0.000 0.000 1500.000

input 6 118.000 0.000 0.000 118.000

LISTING OF PEERS:

peer lambda weight

13 1.000

Results for firm: 14

Technical efficiency = 1.000

Scale efficiency = 1.000 (crs)

PROJECTION SUMMARY:

variable original radial slack projected

value movement movement value

output 1 1455.000 0.000 0.000 1455.000

output 2 336.400 0.000 0.000 336.400

input 1 100000.000 0.000 0.000 100000.000

input 2 4000.000 0.000 0.000 4000.000

input 3 3000.000 0.000 0.000 3000.000

input 4 603.000 0.000 0.000 603.000

input 5 1500.000 0.000 0.000 1500.000

input 6 126.000 0.000 0.000 126.000

LISTING OF PEERS:

peer lambda weight

14 1.000

Results for firm: 15

Technical efficiency = 1.000

Scale efficiency = 1.000 (crs)

PROJECTION SUMMARY:

variable original radial slack projected

value movement movement value

output 1 1571.000 0.000 0.000 1571.000

output 2 352.900 0.000 0.000 352.900

input 1 100000.000 0.000 0.000 100000.000

input 2 4000.000 0.000 0.000 4000.000

input 3 4000.000 0.000 0.000 4000.000

input 4 650.000 0.000 0.000 650.000

input 5 1500.000 0.000 0.000 1500.000

input 6 105.000 0.000 0.000 105.000

LISTING OF PEERS:

peer lambda weight

15 1.000

Results for firm: 16

Technical efficiency = 0.981

Scale efficiency = 0.989 (irs)

PROJECTION SUMMARY:

variable original radial slack projected

value movement movement value

output 1 1502.000 0.000 135.414 1637.414

output 2 349.340 0.000 0.000 349.340

input 1 138000.000 -2573.237 -29514.191 105912.572

input 2 6000.000 -111.880 -1522.605 4365.515

input 3 5000.000 -93.233 -1122.922 3783.845

input 4 707.000 -13.183 0.000 693.817

input 5 1300.000 -24.241 0.000 1275.759

input 6 104.000 -1.939 0.000 102.061

LISTING OF PEERS:

peer lambda weight

20 0.095

17 0.287

15 0.608

2 0.011

Results for firm: 17

Technical efficiency = 1.000

Scale efficiency = 1.000 (crs)

PROJECTION SUMMARY:

variable original radial slack projected

value movement movement value

output 1 1577.000 0.000 0.000 1577.000

output 2 366.730 0.000 0.000 366.730

input 1 120000.000 0.000 0.000 120000.000

input 2 5000.000 0.000 0.000 5000.000

input 3 4040.000 0.000 0.000 4040.000

input 4 700.000 0.000 0.000 700.000

input 5 1000.000 0.000 0.000 1000.000

input 6 107.000 0.000 0.000 107.000

LISTING OF PEERS:

peer lambda weight

17 1.000

Results for firm: 18

Technical efficiency = 0.939

Scale efficiency = 0.924 (drs)

PROJECTION SUMMARY:

variable original radial slack projected

value movement movement value

output 1 1982.000 0.000 0.000 1982.000

output 2 365.200 0.000 0.000 365.200

input 1 130000.000 -7985.602 0.000 122014.398

input 2 6000.000 -368.566 -157.380 5474.054

input 3 5000.000 -307.139 -1515.463 3177.399

input 4 1005.000 -61.735 -22.225 921.041

input 5 1500.000 -92.142 0.000 1407.858

input 6 128.000 -7.863 -1.128 119.009

LISTING OF PEERS:

peer lambda weight

19 0.668

20 0.078

15 0.194

17 0.059

Results for firm: 19

Technical efficiency = 1.000

Scale efficiency = 0.947 (drs)

PROJECTION SUMMARY:

variable original radial slack projected

value movement movement value

output 1 2100.000 0.000 0.000 2100.000

output 2 375.400 0.000 0.000 375.400

input 1 130000.000 0.000 0.000 130000.000

input 2 6000.000 0.000 0.000 6000.000

input 3 3000.000 0.000 0.000 3000.000

input 4 1008.000 0.000 0.000 1008.000

input 5 1500.000 0.000 0.000 1500.000

input 6 130.000 0.000 0.000 130.000

LISTING OF PEERS:

peer lambda weight

19 1.000

Results for firm: 20

Technical efficiency = 1.000

Scale efficiency = 1.000 (crs)

PROJECTION SUMMARY:

variable original radial slack projected

value movement movement value

output 1 2300.000 0.000 0.000 2300.000

output 2 307.500 0.000 0.000 307.500

input 1 110000.000 0.000 0.000 110000.000

input 2 5000.000 0.000 0.000 5000.000

input 3 2000.000 0.000 0.000 2000.000

input 4 1018.000 0.000 0.000 1018.000

input 5 700.000 0.000 0.000 700.000

input 6 69.000 0.000 0.000 69.000

LISTING OF PEERS:

peer lambda weight

20 1.000
